# Red blood cell distribution width for the prediction of outcomes after cardiac arrest

**DOI:** 10.1038/s41598-023-41984-8

**Published:** 2023-09-12

**Authors:** Tabita Urben, Simon A. Amacher, Christoph Becker, Sebastian Gross, Armon Arpagaus, Kai Tisljar, Raoul Sutter, Hans Pargger, Stephan Marsch, Sabina Hunziker

**Affiliations:** 1 Medical Communication and Psychosomatic Medicine, University Hospital Basel, Klingelbergstrasse 23, 4031 Basel, Switzerland; 2 Intensive Care Unit, University Hospital Basel, Basel, Switzerland; 3 Department of Emergency Medicine, University Hospital Basel, Basel, Switzerland; 4 Medical Faculty, University of Basel, Basel, Switzerland

**Keywords:** Prognostic markers, Outcomes research, Cardiovascular diseases

## Abstract

The red blood cell distribution width (RDW) is a routinely available blood marker that measures the variation of the size/volume of red blood cells. The aim of our study was to investigate the prognostic value of RDW in cardiac arrest patients and to assess whether RDW improves the prognostic value of three cardiac arrest-specific risk scores. Consecutive adult cardiac arrest patients admitted to the ICU of a Swiss university hospital were included. The primary outcome was poor neurological outcome at hospital discharge assessed by Cerebral Performance Category. Of 702 patients admitted to the ICU after cardiac arrest, 400 patients (57.0%) survived, of which 323 (80.8%) had a good neurological outcome. Higher mean RDW values showed an independent association with poor neurological outcomes at hospital discharge (adjusted OR 1.27, 95% CI 1.14 to 1.41; p < 0.001). Adding the maximum RDW value to the OHCA- CAHP- and PROLOGUE cardiac arrest scores improved prognostic performance. Within this cohort of cardiac arrest patients, RDW was an independent outcome predictor and slightly improved three cardiac arrest-specific risk scores. RDW may therefore support clinical decision-making.

## Introduction

Sudden cardiac arrest (SCA) is an important cause of death worldwide, and survivors frequently suffer from physical, psychological, and cognitive sequelae^1–4^. As many patients in an early stage after cardiac arrest remain unconscious, prognosis and therapeutic options often need to be discussed with family members who act as surrogate decision-makers^5^. In these early goals of care discussions, expected outcomes, such as neurological outcome, including mortality or disabilities, might have an important impact on decision-making^6^. Therefore, early and reliable prognostic tools may help provide further therapeutic management guidance^6^. So far, some well-validated prognostic models for the prediction of good neurological outcome at hospital discharge after cardiac arrest have been proposed, such as the Out-of-hospital Cardiac Arrest [OHCA] score, the Cardiac Arrest Hospital Prognosis [CAHP] score, and the Prognostication using logistic regression model for unselected adult cardiac arrest patients in the early stages [PROLOGUE] score^7–10^.

A previous study assessed the short-term prognostic value of various routine blood markers, whereas some markers, such as procalcitonin and lactate were able to improve the prognostic performance of a parsimonious clinical prediction model for mortality and neurological outcome^11^. Thus, addition of routine blood markers to clinical prediction models seems to be a promising approach, as these parameters are usually inexpensive and widely available. Among routine blood markers, the red blood cell distribution width (RDW), measures the variation of the size/volume of red blood cells and is often reported routinely as part of the complete blood count^12, 13^. RDW is calculated by dividing the standard deviation (SD) of erythrocytes volume by the mean corpuscular volume (MCV) of erythrocytes expressed as percentage^14, 15^. Commonly, RDW is used in anemia diagnostics, but it recently gained attention for prognostic research by acting as an independent risk factor for death in acute coronary syndrome, lung cancer, acute pulmonary embolism, and unselected intensive care unit [ICU] patients^13, 14, 16–20^. However, only a few studies assessed the prognostic value of RDW in cardiac arrest patients. In the Korean Cardiac Arrest Research Consortium Study [KoCARC study], RDW was found to be independently associated with poor neurological outcome after out-of-hospital cardiac arrest with a good prognostic performance (area under the curve [AUC] 0.63)^21^. Herein, our aim was to validate the results of the KoCARC Study in a large prospective Swiss cohort of cardiac arrest survivors and to investigate if the addition of RDW to three cardiac arrest specific scores (i.e., OHCA-, CAHP-, and PROLOGUE score) improves prediction model performance.

## Methods

### Study setting

Data were prospectively collected on patients in the ongoing COMMUNICATE/PROPHETIC study at a tertiary teaching hospital in Switzerland (University Hospital Basel). Details of the study procedure have been published previously^4, 11, 22–31^. Depending on the patients’ cognitive abilities, the informed consent was given by the patient or a medical guardian. If a medical guardian could not be located, an independent physician acted as the surrogate decision-maker.

The Transparent Reporting of a Multivariable Prediction Model for Individual Prognosis or Diagnosis [TRIPOD] statement guided us through the analysis and reporting of this study^32–34^.

### Participants

Adult patients with return of spontaneous circulation [ROSC] after out-of-hospital [OHCA] or in-hospital [IHCA] cardiac arrest who were admitted to the intensive care unit [ICU] of the University Hospital Basel after cardiac arrest were eligible for inclusion in this study. Monitored patients (e.g., ICU, operating room, cardiac catheterization laboratory) and those who refused informed consent were excluded. Patients’ treatment was based on the standardized local treatment protocol in accordance with the guidelines of the European Resuscitation Council^35, 36^.

### Outcomes

The primary outcome was poor neurological outcome at hospital discharge after cardiac arrest as assessed by the Cerebral Performance Category [CPC] scale^37^. The scale is divided into five categories of functional outcomes: A score of 1 implies good recovery with readmission of everyday life, however, mild neurological or psychological symptoms might be present, a score of 2 implies moderate disability with independence regarding activities of daily living, a score of 3 implies severe disability with dependence on others for activities of daily living, a score of 4 implies any state of coma or persistent vegetative state and a score of 5 includes death or brain death^37^. The secondary outcome was in-hospital mortality. According to previous studies, we dichotomized levels 1 (good recovery) and 2 (moderate disability) as good neurological outcome, whereas levels 3 (severe disability), 4 (vegetative state) and 5 (death) were defined as poor neurological outcome^5, 6^.

### Data collection

The routine blood markers, including RDW, were collected at ICU admission (day 0) and on day 1, 3, 5, 7, as well as on the day of discharge from the ICU. RDW was assessed by the ADVIA hematology system (SIEMENS Healthineers International, Zurich, Switzerland) using impedance with hydrodynamic focusing. RDW is a percentage value with a theoretical range from 0 to 100. For the purpose of this study referred to as ‘RDW value’.

The following data was extracted from the electronic patient record: Demographics (i.e. gender, age), coexisting morbidities (i.e,. coronary artery disease, heart failure, neurologic disease, diabetes, hypertension, chronic obstructive pulmonary disease [COPD], chronic kidney disease, liver cirrhosis and malignancy), resuscitation details (setting of cardiac arrest, observed cardiac arrest, provision of bystander cardiopulmonary resuscitation [CPR], initial rhythm, no-flow time, time until ROSC), etiology of cardiac arrest, clinical and laboratory values (i.e. blood pressure on ICU admission, Glasgow Coma Scale [GCS], red blood cell distribution width, hematocrit, blood pH, lactate) and CPC score at hospital discharge.

The three clinical risk scores used in this study are composed as follows: The OHCA score is calculated by five clinical (no-flow and low-flow interval, initial rhythm) and laboratory values (creatinine, lactate)^7, 31^. The CAHP score contains additional information on resuscitative measures (location of cardiac arrest, epinephrine dosage) and another laboratory parameter (pH) at ICU admission^8, 31^. The PROLOGUE score omits the no-flow time and includes different resuscitation details (unwitnessed arrest, low-flow time, non-shockable rhythm), clinical and laboratory values (age, GCS, pupillary light reflex, adrenaline dose, phosphate, creatinine, hemoglobin, lactate, potassium)^10, 31^.

### Statistical analysis

The statistical analysis was performed by STATA 15.0 using descriptive statistics to specify our cohort with mean and standard deviation (SD) or median and interquartile range (IQR) for continuous variables and frequencies for binary and categorical variables. Continuous variables were visually inspected for normal distribution. A two-sided T-test was used for the analysis of continuous variables, whereas a chi-square test was used for binary and categorical variables. The discrimination of RDW values was calculated by receiver operating characteristics [ROCs] and the corresponding AUC.

To evaluate the association of RDW with our primary and secondary outcomes, univariate and multivariable analyses were performed. The models were adjusted for predefined known confounders of RDW^13, 38–40^ such as age, gender, hematocrit, and comorbidities. Odds ratios [OR] with 95% confidence interval [CI] were calculated using logistic regression for poor neurological outcome and mortality.

The multivariable model was performed by adding the OHCA-, CAHP- and PROLOGUE score to assess the effect of the maximum RDW values over days 0, 1, 3, 5, 7 and ICU discharge on the association with outcomes. In line with the KoCARC study^21^ we calculated 4 quartiles of the mean RDW value with the following ranges: < 13.5, 13.5–14.3, 14.3–15.5, > 15.5.

We investigated specificity and sensitivity using cut-offs regarding poor neurological outcome at hospital discharge. The highest specificity for poor neurological outcome was observed at a maximum RDW value of 13.2, and maximum sensitivity was observed at a maximum RDW value of 16.6. Maximum specificity for mortality at hospital discharge was observed at a maximum RDW value of 13.2, and maximum sensitivity was observed at a maximum RDW value of 17 using the Youden Index.

In addition, RDW cut-offs for the prediction of good neurological outcome at hospital discharge (mean RDW value; cut-off 14.6) and survival to hospital discharge (mean RDW value; cut-off 15.5) as defined by Cheng et al. were validated^41^. The mean RDW values on day 0 and day 7 were compared using a two-tailed paired t-test.

### Ethics approval and consent to participate

The study was permitted by the Ethics Committee of North-western and Central Switzerland (www.eknz.ch) and led by the principles of the Declaration of Helsinki and its amendments.

## Results

### Baseline characteristics

Seven hundred and two consecutive patients admitted to the ICU with ROSC after cardiac arrest were included. Overall, every patient had at least one RDW measurement.

Until hospital discharge 400 patients (57.0%) survived, of which 323 (80.8%) had a good neurological outcome. Table 1 depicts the baseline characteristics stratified by neurological outcome at hospital discharge. The mean age was approximately 65 years, and 506 patients (72.1%) were male. The majority of cases were out-of-hospital cardiac arrests (83.5%), whereas 16.5% were survivors of an in-hospital cardiac arrest. The most frequent cause of cardiac arrest was coronary artery disease (333 patients, 47.8%). Patients with poor neurological outcome at hospital discharge were older (mean ± SD age 67.2 years, ± 13.9 vs. 61.8 years, ± 14.2; *p* < 0.001) and had a higher rate of pre-cardiac arrest comorbidities: COPD (15.3% vs. 5.6%; *p* < 0.001), malignant disease (14.9% vs. 6.8%; *p* < 0.001), neurological disease (19.0% vs. 9.3%; *p* < 0.001), diabetes (24.3% vs. 18.0%; *p* = 0.04) However, survivors with a good neurological outcome more often had pre-cardiac arrest evidence of coronary artery disease (64.1% vs. 52.9%; *p* = 0.003). Patients with a poor neurological outcome at hospital discharge had a longer mean no-flow time (4.71 min vs. 1.18 min; *p* < 0.001) and less often received bystander CPR (60.3% vs. 83.9%; *p* < 0.001).

**Table 1:** Baseline characteristics.

Factor	All patients (N = 702)	Good neurological outcome: CPC 1–2 (N = 323)	Poor neurological outcome: CPC 3–5 (N = 379)	*p*-value
**Sociodemographics**				
Age [years], mean (SD)	64.7 (14.3)	61.8 (14.2)	67.2 (13.9)	< *0.001*
Male gender, n (%)	506 (72.1%)	255 (78.9%)	251 (66.2%)	< *0.001*
**Comorbidities**				
Coronary artery disease, n (%)	407 (58.1%)	207 (64.1%)	200 (52.9%)	*0.003*
Congestive heart failure, n (%)	100 (14.3%)	38 (11.8%)	62 (16.4%)	0.08
COPD, n (%)	76 (10.8%)	18 (5.6%)	58 (15.3%)	< *0.001*
Liver disease, n (%)	19 (2.7%)	5 (1.5%)	14 (3.7%)	0.1
Hypertension, n (%)	362 (51.6%)	169 (52.3%)	193 (51.1%)	0.76
Diabetes, n (%)	150 (21.4%)	58 (18.0%)	92 (24.3%)	*0.04*
Chronic kidney disease, n (%)	95 (13.6%)	37 (11.5%)	58 (15.3%)	0.15
Malignant disease, n (%)	78 (11.1%)	22 (6.8%)	56 (14.9%)	< *0.001*
Neurological disease, n (%)	102 (14.6%)	30 (9.3%)	72 (19.0%)	< *0.001*
**Resuscitation measures**				
No-flow Time [min], mean (SD)	3.01 (5.27)	1.18 (2.66)	4.71 (6.41)	< *0.001*
Time until ROSC [min], mean (SD)	22.02 (18.38)	16.19 (13.75)	27.41 (20.38)	< *0.001*
Observed cardiac arrest, n (%)	572 (81.6%)	293 (90.7%)	279 (73.8%)	< *0.001*
Bystander CPR, n (%)	499 (71.2%)	271 (83.9%)	228 (60.3%)	< *0.001*
**Setting of cardiac arrest**				< *0.001*
At home, n (%)	258 (37.2%)	92 (29.0%)	166 (44.1%)	
Public, n (%)	321 (46.3%)	173 (54.6%)	148 (39.4%)	
IHCA, n (%)	114 (16.5%)	52 (16.4%)	62 (16.5%)	
**Cause of cardiac arrest**				< *0.001*
Coronary artery disease, n (%)	333 (47.8%)	195 (61.3%)	138 (36.4%)	
Primary arrhythmia, n (%)	100 (14.3%)	53 (16.7%)	47 (12.4%)	
Other/unknown, n (%)	264 (37.9%)	70 (22.0%)	194 (51.2%)	
**Initial rhythm**				< *0.001*
Ventricular fibrillation, n (%)	335 (47.9%)	217 (67.2%)	118 (31.3%)	
Ventricular tachycardia, n (%)	33 (4.7%)	18 (5.6%)	15 (4.0%)	
Asystole, n (%)	113 (16.1%)	14 (4.3%)	99 (26.3%)	
Pulseless electrical activity, n (%)	159 (22.7%)	36 (11.1%)	123 (32.6%)	
Unknown, n (%)	60 (8.6%)	38 (11.8%)	22 (5.8%)	
**Initial Status at ICU admission**				
Total GCS, mean (SD)	6 (4)	7 (5)	4 (3)	< *0.001*
Systolic blood pressure [mmHg], mean (SD)	115 (25)	116 (22)	115 (28)	0.45
Diastolic blood pressure [mmHg], mean (SD)	67 (17)	69 (15)	65 (18)	< *0.001*
Mean arterial pressure [mmHg], mean (SD)	82 (18)	84 (16)	81 (19)	*0.01*
Initial pH, mean (SD)	7.21 (.166)	7.26 (.135)	7.17 (.175)	< *0.001*
Initial lactate [mmol/l], mean (SD)	6.45 (4.41)	4.66 (3.5)	7.89 (4.55)	< *0.001*
Hematocrit [l/l], mean (SD)	.395 (.0697)	.402 (.062)	.39 (.0751)	*0.03*
**Diagnostic measures**				
RDW max [%], mean (SD)	14.77 (1.96)	14.30 (1.64)	15.18 (2.11)	< *0.001*
RDW max [%], median	14.30 (13.50, 15.50)	13.80 (13.20, 15.00)	14.60 (13.80, 15.90)	< *0.001*

*COPD* chronic obstructive pulmonary disease, *CPC* cerebral performance category scale, *CPR* cardiopulmonary resuscitation, *ICU* intensive care unit, *IHCA* in-hospital cardiac arrest, *GCS* glasgow coma scale; *n* number, *RDW* red cell distribution width, *ROSC* return of spontaneous circulation, *SD* standard deviation.

Significant values are in Italic.

### Association of RDW and risk scores with neurological outcome at hospital discharge

**Figure 1:**
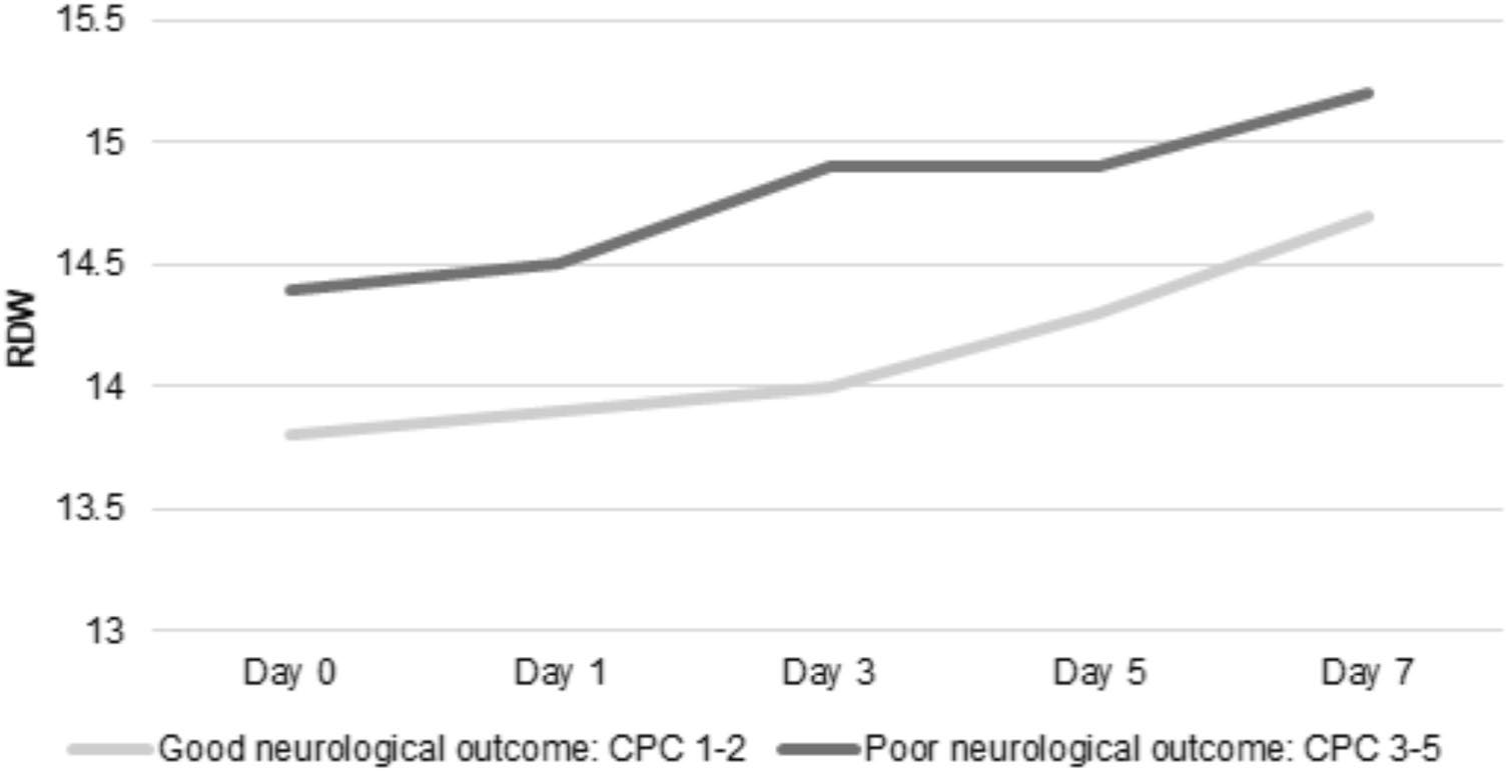
The dynamic of mean RDW values during ICU stay, stratified by the primary outcome (CPC). CPC Cerebral performance category scale; RDW Red blood cell distribution width.

Higher mean RDW values were independently associated with poor neurological outcome at hospital discharge (mean, ± SD 15 ± 2 vs 14 ± 2, adjusted OR 1.27, 95% CI 1.14–1.41; *p* < 0.001; Table 2). Patients with poor neurological outcome at hospital discharge had higher mean RDW values on day 0, 1, 3 and 5. The highest mean RDW difference between patients with poor and good neurological outcome, as illustrated in Fig. 1, was found on day 3 (mean ± SD 14.9 ± 2.02 vs. 14 ± 1.51, adjusted OR 1.28, 95% CI 1.10–1.49; *p* = 0.002).

**Table 2:** Logistic regression analysis.

	Good neurological outcome: CPC 1–2 (N = 323)	Poor neurological outcome: CPC 3–5 (N = 379)	*p*-value	Univariate	Multivariable* adjusted	RDW and OHCA score: multivariable* adjusted	RDW and CAHP score: multivariable* adjusted	RDW and PROLOGUE score: multivariable* adjusted	Univariate	Multivariable* adjusted
				OR (95%CI), p	OR (95%CI), p	OR (95%CI), p	OR (95%CI), p	OR (95%CI), p	AUC	AUC combined with RDW Max
**A Primary outcome: Neurological outcome at hospital discharge**										
RDW Max, mean (SD)	14 (2)	15 (2)	< *0.001*	1.31 (1.19, 1.44), *p* < *0.001*	1.27 (1.14, 1.41), *p* < *0.001*	1.19 (1.06, 1.33), *p* = *0.002*	1.19 (1.07, 1.34), *p* = *0.002*	1.2 (1.08, 1.33), *p* = *0.001*	0.65	n.a
RDW Max, median (IQR)	14 (13, 15)	15 (14, 16)	< *0.001*							
Quartiles of RDW Max., n (%)	123 (38.1%)	72 (19.0%)	< *0.001*	*(Ref 1)*	*(Ref 1)*	*(Ref 1)*	*(Ref 1)*	*(Ref 1)*	n.a	n.a
2nd quartile, n (%)	90 (27.9%)	87 (23.0%)		1.65 (1.09, 2.5),* p* = *0.02*	1.4 (0.91, 2.17), *p* = *0.13*	1.21 (0.72, 2.03), *p* = *0.48*	1.09 (0.65, 1.84), *p* = *0.75*	1.04 (0.64, 1.68), *p* = *0.89*		
3rd quartile, n (%)	60 (18.6%)	104 (27.4%)		2.96 (1.92, 4.56), *p* < *0.001*	2.41 (1.53, 3.8), *p* < *0.001*	1.91 (1.11, 3.31), *p* = *0.02*	1.88 (1.09, 3.25), *p* = *0.02*	1.58 (0.95, 2.62), *p* = *0.08*		
4th quartile, n (%)	50 (15.5%)	116 (30.6%)		3.96 (2.55, 6.16),* p* < *0.001*	3.14 (1.91, 5.15), *p* < *0.001*	2.32 (1.3, 4.14), *p* = *0.005*	2.17 (1.21, 3.9), *p* = *0.01*	2.33 (1.36, 4.01),* p* = *0.002*		
RDW Day 0, mean (SD)	13.8 (1.36)	14.4 (1.87)	< *0.001*	1.29 (1.16, 1.44), *p* < *0.001*	1.18 (1.05, 1.33), *p* = *0.006*	1.1 (0.97, 1.26), *p* = *0.13*	1.11 (0.97, 1.26), *p* = *0.12*	1.12 (0.99, 1.27),* p* = *0.07*		
RDW Day 1, mean (SD)	13.9 (1.34)	14.5 (1.81)	< *0.001*	1.35 (1.2, 1.52), *p* < *0.001*	1.26 (1.1, 1.44), *p* = *0.001*	1.17 (1.02, 1.35),* p* = *0.03*	1.19 (1.03, 1.36),* p = 0.02*	1.19 (1.05, 1.36), *p* = *0.009*		
RDW Day 3, mean (SD)	14 (1.51)	14.9 (2.02)	< *0.001*	1.37 (1.19, 1.57), *p* < *0.001*	1.28 (1.1, 1.49),* p* = *0.002*	1.24 (1.06, 1.45), *p* = *0.007*	1.25 (1.07, 1.46), *p* = *0.005*	1.26 (1.08, 1.46), *p* = *0.003*		
RDW Day 5, mean (SD)	14.3 (1.65)	14.9 (1.8)	*0.007*	1.23 (1.05, 1.44),* p* = *0.009*	1.2 (1, 1.44), *p* = *0.06*	1.16 (0.95, 1.41), *p* = *0.15*	1.17 (0.96, 1.42),* p* = *0.11*	1.18 (0.98, 1.42), *p* = *0.08*		
RDW Day 7, mean (SD)	14.7 (1.8)	15.2 (1.74)	*0.063*	1.19 (0.99, 1.44),* p* = *0.07*	1.18 (0.95, 1.46), *p* = *0.14*	1.11 (0.89, 1.4),* p* = *0.34*	1.15 (0.93, 1.44), *p* = *0.21*	1.17 (0.94, 1.45), *p* = *0.16*		
RDW at ICU discharge, mean (SD)	14.1 (1.6)	15.1 (2.13)	< *0.001*	1.39 (1.26, 1.54),* p* < *0.001*	1.33 (1.19, 1.49), *p* < *0.001*	1.23 (1.09, 1.39),* p* = *0.001*	1.23 (1.09, 1.39), *p* = *0.001*	1.25 (1.11, 1.4), *p* < *0.001*		
OHCA, mean (SD)	8 (18)	30 (18)	< *0.001*	1.07 (1.06, 1.08),* p* < *0.001*	1.07 (1.06, 1.09), *p* < *0.001*	n.a	n.a	n.a	0.82	0.83, *p* < *0.05*
CAHP, mean (SD)	128 (39)	185 (42)	< *0.001*	1.03 (1.03, 1.04),* p* < *0.001*	1.03 (1.03, 1.04), *p* < *0.001*				0.83	0.84, *p* = *0.03*
PROLOGUE, mean (SD)	231 (103)	332 (106)	< *0.001*	1.01 (1.01, 1.01), *p* < *0.001*	1.01 (1.01, 1.01), *p* < *0.001*				0.75	0.77, *p* = *0.02*

	Survivors (N = 400)	Non-Survivors (N = 302)	*p*-value	Univariate	Multivariable* adjusted	RDW and OHCA score: multivariable* adjusted	RDW and CAHP score: multivariable* adjusted	RDW and PROLOGUE score: multivariable* adjusted	Univariate	Multivariable* adjusted
				OR (95%CI), p	OR (95%CI), p	OR (95%CI), p	OR (95%CI), p	OR (95%CI), p	AUC	AUC combined with RDW Max
**B Secondary endpoint: Mortality at hospital discharge**										
RDW Max, mean (SD)	14.5 (1.87)	15.1 (2.04)	< *0.001*	1.16 (1.07, 1.25), *p* < *0.001*	1.1 (1.01, 1.21), *p* = *0.04*	1.03 (0.93, 1.14), *p* = *0.59*	1.03 (0.92, 1.14), *p* = *0.63*	1.04 (0.94, 1.15), *p* = *0.46*	0.61	n.a
RDW Max, median (IQR)	14 (13, 15)	15 (14, 16)	< *0.001*							
Quartiles of RDW Max, n (%)	135 (33.8%)	60 (19.9%)	< *0.001*	*(Ref 1)*	*(Ref 1)*	*(Ref 1)*	*(Ref 1)*	*(Ref 1)*	n.a	n.a
2nd quartile, n (%)	106 (26.5%)	71 (23.5%)		1.51 (0.98, 2.31), *p* = *0.06*	1.32 (0.85, 2.07), *p* = *0.22*	1.14 (0.67, 1.93), *p* = *0.63*	1.05 (0.61, 1.8), *p* = *0.85*	0.91 (0.54, 1.52),* p* = *0.71*		
3rd quartile, n (%)	80 (20.0%)	84 (27.8%)		2.36 (1.53, 3.64), *p* < *0.001*	1.97 (1.25, 3.1), *p* = *0.003*	1.42 (0.83, 2.45), *p* = *0.2*	1.47 (0.85, 2.53), *p* = *0.17*	1.13 (0.67, 1.91), *p* = *0.65*		
4th quartile, n (%)	79 (19.8%)	87 (28.8%)		2.48 (1.61, 3.81), *p* < *0.001*	1.81 (1.12, 2.94),* p* = *0.02*	1.19 (0.67, 2.1), *p* = *0.55*	1.11 (0.62, 1.98), *p* = *0.73*	1.19 (0.69, 2.06), *p* = *0.54*		
RDW Day 0, mean (SD)	13.9 (1.55)	14.4 (1.82)	< *0.001*	1.17 (1.07, 1.29), *p* = *0.001*	1.08 (0.98, 1.21), *p* = *0.13*	1.01 (0.89, 1.14), *p* = *0.9*	1.01 (0.89, 1.14), *p* = *0.93*	1.03 (0.91, 1.15), *p* = *0.68*		
RDW Day 1, mean (SD)	14 (1.59)	14.5 (1.66)	*0.001*	1.17 (1.06, 1.3), *p* = *0.002*	1.09 (0.97, 1.22), *p* = *0.16*	1 (0.87, 1.14),* p* = *0.98*	1.02 (0.89, 1.16), *p* = *0.79*	1.03 (0.91, 1.17),* p* = *0.63*		
RDW Day 3, mean (SD)	14.3 (1.77)	14.8 (1.91)	*0.004*	1.18 (1.05, 1.32), *p* = *0.005*	1.1 (0.96, 1.26),* p* = *0.16*	1.07 (0.93, 1.24), *p* = *0.32*	1.08 (0.93, 1.24), *p* = *0.31*	1.08 (0.94, 1.24), *p* = *0.25*		
RDW Day 5, mean (SD)	14.5 (1.75)	14.8 (1.73)	*0.24*	1.09 (0.94, 1.27), *p* = *0.24*	1.09 (0.92, 1.3), *p* = *0.33*	1.07 (0.89, 1.28), *p* = *0.5*	1.07 (0.89, 1.28), *p* = *0.5*	1.08 (0.91, 1.29), *p* = *0.37*		
RDW Day 7, mean (SD)	14.9 (1.86)	15.1 (1.59)	*0.51*	1.07 (0.88, 1.3), *p* = *0.50*	1.04 (0.83, 1.31), *p* = *0.74*	1.02 (0.8, 1.29), *p* = *0.89*	1.03 (0.81, 1.31), *p* = *0.83*	1.03 (0.81, 1.3), *p* = *0.84*		
RDW at ICU discharge, mean (SD)	14.3 (1.76)	15.1 (2.14)	< *0.001*	1.26 (1.16, 1.38), *p* < *0.001*	1.19 (1.07, 1.32),* p* = *0.001*	1.11 (0.99, 1.25), *p* = *0.07*	1.11 (0.99, 1.24), *p* = *0.09*	1.12 (1, 1.25), *p* = *0.04*		
OHCA, mean (SD)	11 (19)	33 (17)	< *0.001*	1.08 (1.07, 1.09), *p* < *0.001*	1.07 (1.06, 1.09), *p* < *0.001*	n.a	n.a	n.a	0.81	0.81, *p* = *0.36*
CAHP, mean (SD)	135 (42)	190 (40)	< *0.001*	1.03 (1.03, 1.04), *p* < *0.001*	1.03 (1.03, 1.04), *p* < *0.001*				0.83	0.83,* p* = *0.58*
PROLOGUE, mean (SD)	236 (102)	350 (101)	< *0.001*	1.01 (1.01, 1.01), *p* < *0.001*	1.01 (1.01, 1.01), *p* < *0.001*				0.78	0.79, *p* = *0.31*

*AUC* area under curve, *CAHP* cardiac arrest hospital prognosis score, *CI* confidence interval, *COPD* chronic obstructive pulmonary disease, *CPC* cerebral performance category scale, *ICU* intensive care unit; *n* number, *n.a.* not available, *OHCA* out-of-hospital cardiac arrest score, *OR* odds ratio, *PROLOGUE* prognostication using logistic regression model for unselected adult cardiac arrest patients in the early stages, *RDW* red cell distribution width

*Adjusted for age, gender, hematocrit, comorbidities.

All three risk scores were associated with poor neurological outcome with AUCs for the OHCA-, CAHP- and PROLOGUE score of 0.82, 0.83 and 0.75. When adding the maximum RDW values to these scores, the prognostic performance of all scores slightly improved: OHCA score from AUC 0.82 to 0.83; *p* = 0.047; CAHP score from AUC 0.83 to 0.84; *p* = 0.03 and PROLOGUE score from AUC 0.75 to 0.77; *p* = 0.02.

### Association of RDW with mortality at hospital discharge

Regarding secondary outcome, higher mean RDW values were independently associated with mortality at hospital discharge (mean ± SD 15.1 ± 2.04 vs 14.5 ± 1.87, adjusted OR 1.1, 95% CI 1.01–1.21; *p* = 0.04). Further, the third (RDW values 14.3–15.5) and fourth (RDW values < 15.5) quartile were independently associated with mortality at hospital discharge (adjusted OR 1.97, 95% CI 1.25–3.1; *p* = 0.003 and OR 1.81, 95% CI 1.12–2.94; *p* = 0.02).

The maximum RDW values did not improve the prognostic performance of the OHCA-, CAHP- and PROLOGUE score regarding mortality.

**Figure 2:**
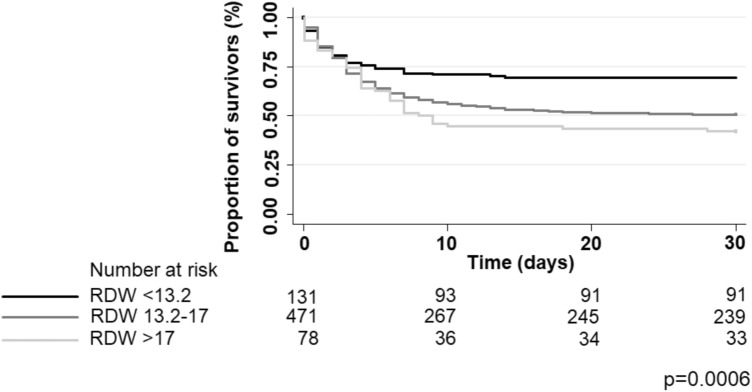
Kaplan–Meier-curve for 30-day all-cause mortality stratified according to low (< 13.2), moderate (13.2–17) and high (> 17) RDW values. RDW Red blood cell distribution width.

A Kaplan–Meier-curve for 30-day all-cause-mortality stratified by the two different optimal cut-offs of mean maximum RDW values (< 13.2 and > 17) calculated by Youden Index is shown in Fig. 2.

### RDW dynamic over time

The mean RDW values increased over time, with the highest values observed at day 7. Overall a significant difference between day 0 and day 7 (14.1 vs. 14.9, mean difference 0.8, 95% CI 0.61–1.04, *p* < 0.001) was observed. This could also be observed when stratifying the increase of RDW values over time according to the primary outcome: Day 0 vs. day 7 in patients with poor neurological outcome (14.2 vs. 15.2, mean difference 1.0, 95% CI 0.74–1.33, *p* < 0.001) and day 0 vs. day 7 in patients with good neurological outcome (14.0 vs. 14.7, mean difference 0.7, 95% CI 0.32–0.94, *p* = 0.001), respectively.

### Sensitivity and specificity

We also assessed the sensitivity and specificity of maximum RDW values at different cut-offs (Table 3). For poor neurological outcome, the optimal cut-off at a maximum RDW value (Youden Index) of 13.2 had a sensitivity of 91% and specificity of 23.5%. A cut-off at a maximum RDW value of 16.6 resulted in a sensitivity of 20.6% and a specificity of 89.8%.

**Table 3:** Sensitivity and specificity.

		Poor neurological outcome: CPC 3–5		Mortality at hospital discharge	
		RDW Max* cut-off 13.2	RDW Max* cut-off 16.6	RDW Max* cut-off 13.2	RDW Max* cut-off 17
Prevalence	Pr(A)	54.00%	54.00%	43.00%	43.00%
Sensitivity	Pr(+ A)	91.00%	20.60%	91.10%	15.20%
Specificity	Pr(-N)	23.50%	89.80%	20.80%	90.00%
Likelihood ratio ( +)	Pr(+ A)/Pr(+ N)	1.19	2.01	1.15	1.52
Likelihood ratio (-)	Pr(-A)/Pr(-N)	0.38	0.88	0.43	0.94
Odds ratio	LR( +)/LR(-)	3.12	2.28	2.67	1.62
Positive predictive value	Pr(A +)	58.30%	70.30%	46.50%	53.50%
Negative predictive value	Pr(N-)	69.10%	49.10%	75.50%	58.40%

*CPC* cerebral performance category scale, *RDW* red cell distribution width.

*RDW Max indicates the mean of each patients maximum RDW value.

For mortality at hospital discharge, a cut-off at a maximum RDW value of 13.2 had a sensitivity of 91.1% and a specificity of 20.8%. A cut-off at a maximum RDW value of 17 had a sensitivity and specificity of 15.2% and 90%.

The proposed cut-offs by Cheng et al.^41^ had a sensitivity of 52% and specificity of 70% for poor neurological outcome, and a sensitivity of 30.8% and specificity of 79.2% for mortality (Supplementary Table S1).

## Discussion

In our cohort of cardiac arrest patients, RDW is an independent predictor of poor neurological outcome at hospital discharge, with mean RDW values on day three showing the strongest association. These findings persisted when adjusting the results for known RDW confounders such as age, gender, hematocrit, and comorbidities^13, 38–40^. Also, a higher mean RDW value was found to be an independent predictor of mortality at hospital discharge.

As an inexpensive, routinely available blood marker, RDW might be a promising prognostic factor to support clinical decision-making. It could help to stewardship the management in a early course after cardiac arrest, especially if patient are unconscious and thus difficult to be neurologically assessed. Also, when integrated into established clinical prediction models such as OHCA-, CAHP- or PROLOGUE score, it might improve their predictive value.

Several studies found RDW to be an independent risk factor for death in acute coronary syndrome, lung cancer, acute pulmonary embolism, and unselected ICU patients^13, 14, 16–20^. Many factors are associated with an increase in RDW, such as age, sex, anemia, inflammatory markers (e.g. C-reactive protein [CRP]), metabolic syndromes, or blood transfusion^13, 38, 39, 42–44^. As cardiac arrest patients usually face a relevant phase of hypoxemia, one possible explanation for elevated RDW levels are hypoxia-induced elevated erythropoietin levels which also occur in patients with other acute conditions such as pneumonia, pulmonary embolism, pneumothorax, cardiac disease or sepsis^45^. This might also explain the higher mean RDW values in patients with non-observed cardiac arrest and a consecutively longer no-flow time. Accordingly, these criteria are part of modern risk prediction scores for neurological outcome after cardiac arrest^9^.

RDW was also elevated in patients with chronic kidney disease^38^ and might be an indication for an underlying inflammatory state^38, 43, 46^. However, it is unclear if in our cohort the elevated RDW is an acute response to the cardiac arrest, to pre-existing comorbidities or to ICU treatment.

Our results are mostly in line with previous studies investigating the prognostic performance of RDW in cardiac arrest patients where RDW was found to be an independent predictor of poor neurological outcome and mortality at hospital discharge or 30-days after hospital discharge^21, 41, 47^. Differences in study setting, study population and severity of illness may cause variations in results. Generally, two studies collected blood samples immediately after admission to the emergency department whereas we only included patients who survived until ICU admission^21, 47^. Woo et al. reported much more patients with poor neurological outcome (70.6%), due to cultural and legal differences, as in Korea withdrawal of life-sustaining therapy was not allowed until 2018^10, 48^. Also, patients in their cohort received less bystander CPR (54.1%) compared to our cohort (71.2%) and the no-flow time as a marker for hypoxemic burden was not reported^21^. Also, the pre-existing cut-offs from the KoCARC-study were not adequate for our cohort which may be attributed to different analytic techniques and the resulting lack of harmonization^12, 15^. Consistent with previous studies, we found RDW values to be independent predictors of 30-day mortality^21, 47^. However, in our cohort the prognostic value regarding mortality was not as strong. This may be due to the previously mentioned differences in study setting and population.

Importantly, there was a slight improvement of prognostic value of existing cardiac arrest scoring systems (OHCA-, CAHP- and PROLOGUE score) by adding RDW. Thus, RDW brings a benefit as a marker of no-flow time, since this clinical parameter is often missing or unprecise^31^. Previous studies have found RDW to be elevated with age^49, 50^. Also, in our study, RDW was associated with increasing age, however, RDW remained an independent predictor of poor neurological outcome even after adjusting the statistical model for age.

In our cohort, we found an increase in RDW values during the ICU stay. Only few studies have looked at the progression of RDW over time after an initial event. In an observational study, looking at the relationship between RDW and long-term neurological outcome after cardiac arrest, RDW did not increase over time, however, the sampling time was restricted to the first 72 h after cardiac arrest, which prohibits conclusions for longer than 72 h post-cardiac arrest^51^. In a cohort of patients with post-hypoxia (e.g., heart failure, pneumonia, atelectasis, pulmonary embolism, pneumothorax, and sepsis), RDW reached its maximum level within one month after the index event and remained elevated for three months in total^45^.

This study has several strengths. First, the presented data is the result of a large and well-established cardiac arrest cohort over several years. Second, the analysis and reporting followed the TRIPOD statement. Third, the score values were calculated by the study team and were not communicated to the treating physicians, which reduces the concern of self-fulfilling prophecies, a common issue in unblinded prognostic research in cardiac arrest patients^52–54^.

This study also has several limitations. First, our setting of an observational, single-center study limits the transfer to cohorts from other regions or countries. Second, we could not assess causes for elevated RDW and / or death, so there may be nondependent causes. Third, the lack of harmonization in RDW measurement technique may influence the different cut-offs and render our results difficult to compare to other studies^15^. Finally, we did not collect any data about blood transfusions or nutritional state which could also increase RDW values^38, 44^.

## Conclusion

In our prospective cohort of unselected adult cardiac arrest patients, RDW was an independent predictor of poor neurological outcome at hospital discharge. Therefore, RDW could act as an inexpensive and easily available prognostic marker in cardiac arrest patients. Further studies should focus on the prognostic value of RDW for neurological long-term outcome.

### Supplementary Information

Supplementary Information.

## Data Availability

The datasets used and/or analyzed during the current study are available from the corresponding author on reasonable request.

## Abbreviations

AUC: Area under the curve
CAHP: Cardiac arrest hospital prognosis
CI: Confidence interval
COMMUNICATE: Communication in out-of hospital cardiac arrest events
COPD: Chronic obstructive pulmonary disease
CPC: Cerebral performance category
CPR: Cardiopulmonary resuscitation
CRP: C-reactive protein
EEG: Electroencephalogram
EKNZ: Ethikkommission Nordwest- und Zentralschweiz (Ethics committee of north-western and central Switzerland)
GCS: Glasgow coma scale
ICU: Intensive care unit
IHCA: In-hospital cardiac arrest
KoCARC: Korean Cardiac Arrest Research Consortium
MCV: Mean corpuscular volume
NPV: Negative predictive value
OHCA: Out-of-hospital cardiac arrest
OR: Odds ratio
PPV: Positive predictive value
PROLOGUE: Prognostication using logistic regression model for unselected adult cardiac arrest patients in the early stages
PROPHETIC: Prognostication of outcome in patients with out-of hospital cardiac arrest hospitalized in intensive care
RDW: Red blood cell distribution width
ROC: Receiver operating characteristic curve
ROSC: Return of spontaneous circulation
SCA: Sudden cardiac arrest
SD: Standard deviation
TRIPOD: Transparent reporting of a multivariable prediction model for individual prognosis or diagnosis
