# Supplementary material for: Red blood cell distribution width for the prediction of outcomes after cardiac arrest
Source: Sci Rep. 2023 Sep 12;13:15081. doi: 10.1038/s41598-023-41984-8 (PMC10497505; doi:10.1038/s41598-023-41984-8)
Supplement: Supplementary file 1 — Supplementary Information. [file 41598_2023_41984_MOESM1_ESM.pdf]

# Supplementary Information

# Red blood cell distribution width for the prediction of outcomes after cardiac arrest

Tabita Urben, MMed[1*]; Simon A. Amacher, MD[1,2*]; Christoph Becker, MD[1,3]; Sebastian Gross, MSc[1]; Armon Arpagaus, MD[1]; Kai Tisljar, MD[2]; Raoul Sutter, MD[2,4]; Hans Pargger, MD[2,4]; Stephan Marsch, MD PhD[2,4] and Sabina Hunziker, MD MPH[1,4]

[1] Medical Communication and Psychosomatic Medicine, University Hospital Basel, Basel, Switzerland.

[2] Intensive Care Unit, University Hospital Basel, Basel, Switzerland.

[3] Department of Emergency Medicine, University Hospital Basel, Basel, Switzerland.

[4] Medical Faculty, University of Basel, Basel, Switzerland.

* These authors contributed equally to this work and should be considered equal first authors

| Table S1 – Sensitivity and Specificity (cut-offs by Cheng et al*) | | | |
|---|---|---|---|
| | | **Poor neurological outcome: CPC 3-5**<br>**RDW Max** cut-off 14.6** | **Mortality at hospital discharge**<br>**RDW Max** cut-off 15.5** |
| Prevalence | Pr(A) | 54.00% | 43.00% |
| | | | |
| Sensitivity | Pr(+A) | 52.00% | 30.80% |
| Specificity | Pr(-N) | 70.00% | 79.20% |
| | | | |
| Likelihood ratio (+) | Pr(+A)/Pr(+N) | 1.73 | 1.48 |
| Likelihood ratio (-) | Pr(-A)/Pr(-N) | 0.69 | 0.87 |
| Odds ratio | LR(+)/LR(-) | 2.52 | 1.7 |
| Positive predictive value | Pr(A+) | 67.00% | 52.80% |
| Negative predictive value | Pr(N-) | 55.40% | 60.30% |

*Cheng, Y. *et al.* Associations between red cell distribution width and outcomes of adults with in-hospital cardiac arrest: A retrospective study. *Medicine (Baltimore)* **101**, e28750 (2022). https://doi.org:10.1097/md.0000000000028750
**RDW Max picks the mean of the maximum RDW value from the cohort

**CPC** Cerebral performance category scale; **RDW** Red cell distribution width
